# Art of imaging: radiology evolution

**DOI:** 10.1093/radadv/umag023

**Published:** 2026-07-07

**Figure:**
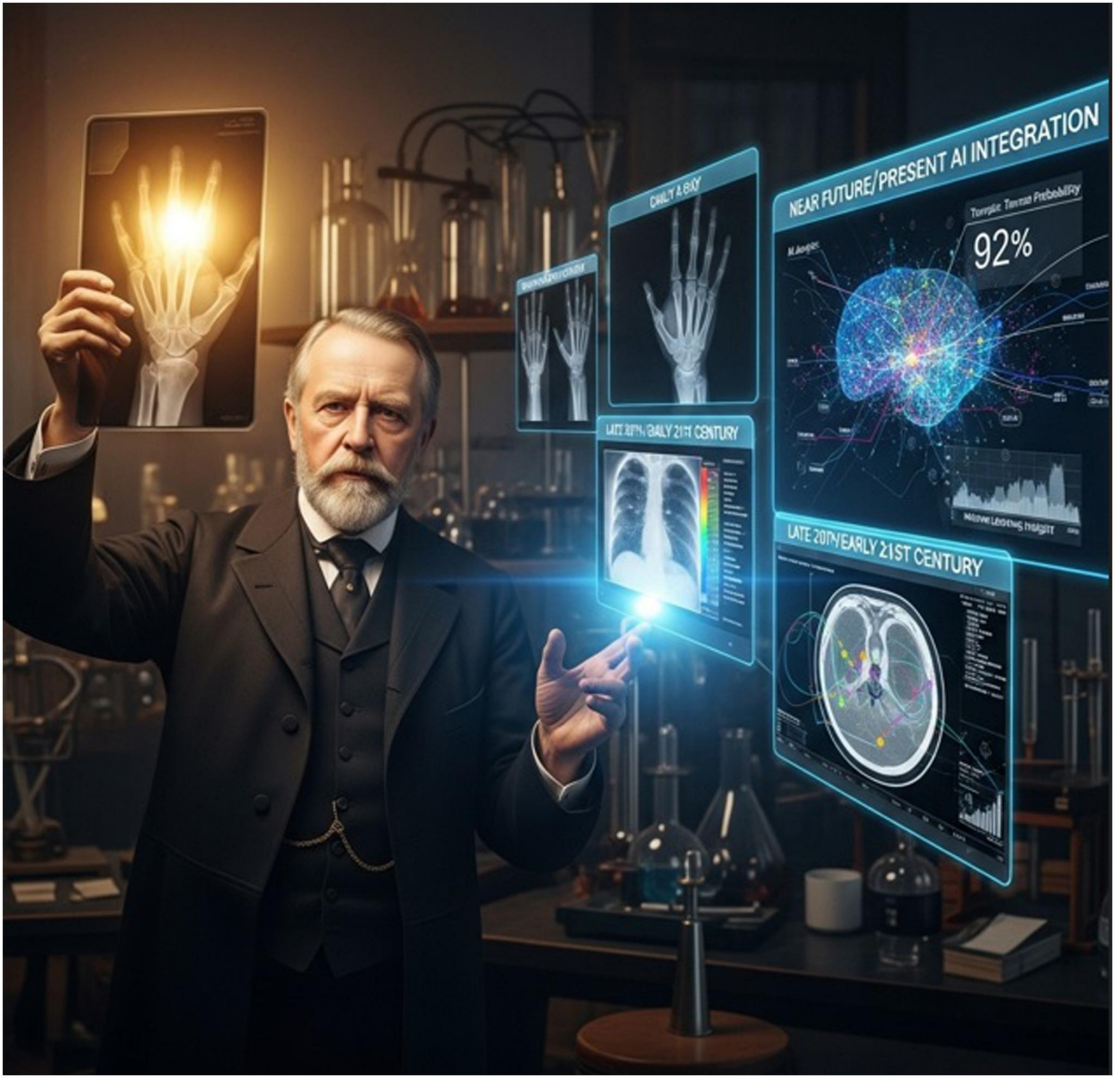
This image represents the transformation of radiology from the invention of X-rays to the present by embracing new technologies.

**Figure:**
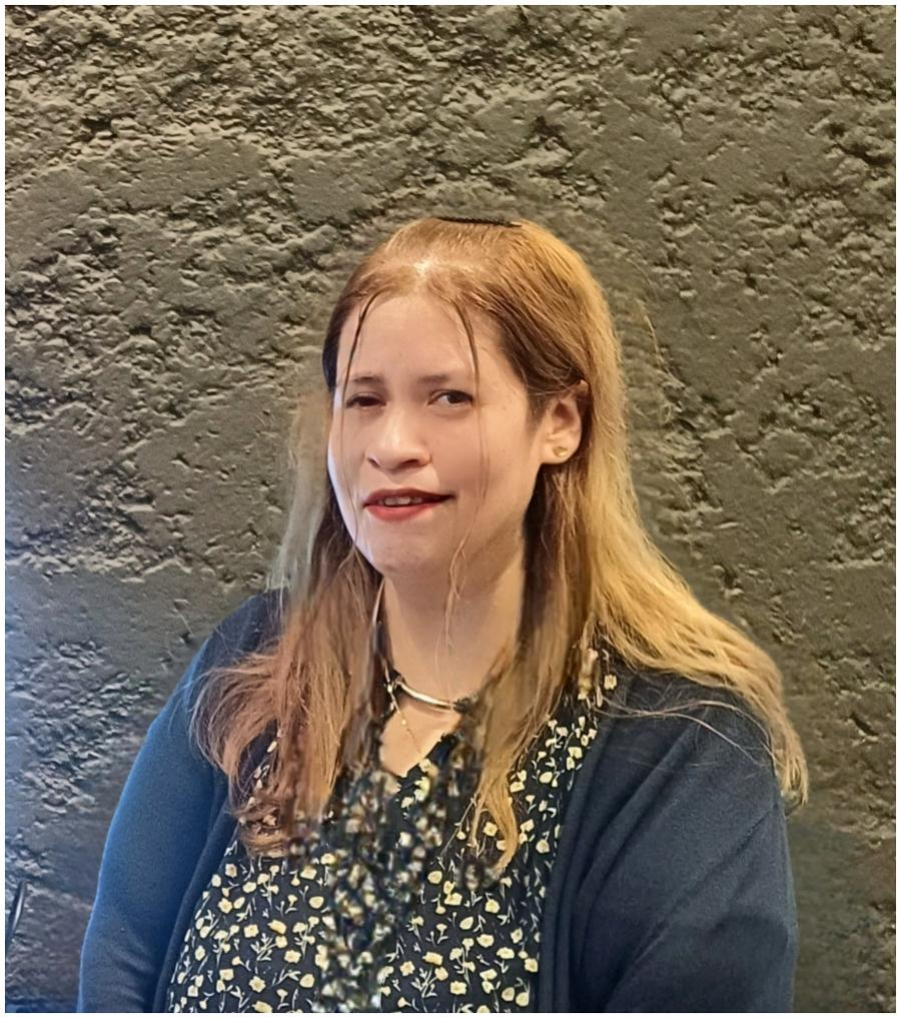


**Norma Patricia Arroyo Lopez, MD** 

Radiologist in Central South High Specialty PEMEX Hospital, Mexico City, Mexico.

